# Autophagy: New Insights into Its Roles in Cancer Progression and Drug Resistance

**DOI:** 10.3390/cancers12103005

**Published:** 2020-10-16

**Authors:** Steffan T. Nawrocki, Wei Wang, Jennifer S. Carew

**Affiliations:** 1 University of Arizona Cancer Center, Tucson, AZ 85724, USA; jcarew@email.arizona.edu; 2 Division of Translational and Regenerative Medicine, Department of Medicine, University of Arizona, Tucson, AZ 85721, USA; 3 Department of Pharmacology and Toxicology, University of Arizona, Tucson, AZ 85721, USA; wwang@pharmacy.arizona.edu; 4 Arizona Center for Drug Discovery, Tucson, AZ 85721, USA

**Keywords:** autophagy, drug resistance, cancer, ROC-325, lysosome

## Abstract

Autophagy is a mechanism of lysosomal proteolysis that is utilized to degrade damaged organelles, proteins, and other cellular components. Although key studies demonstrate that autophagy functions as a mechanism of tumor suppression via the degradation of defective pre-malignant cells, autophagy can also be used as a mechanism to break down cellular components under stress conditions to generate the required metabolic materials for cell survival. Autophagy has emerged as an important mediator of resistance to radiation, chemotherapy, and targeted agents. This series of articles highlight the role of autophagy in cancer progression and drug resistance and underscores the need for new and more effective agents that target this process.

Autophagy is an evolutionarily conserved protein degradation process that is characterized by the formation of double-membraned vesicles (autophagosomes) that envelop bulk cellular material and/or organelles. Autophagosomes subsequently fuse with lysosomes and the degradation of their cargo is mediated by lysosomal proteases [1,2]. Autophagy is an essential cellular process that degrades damaged organelles, long-lived proteins, and recycles cellular components to generate the metabolic building blocks required for cell survival. Induction of autophagy has been reported to play both pro-death and pro-survival roles depending upon the specific cellular context. Pro-death-mediated autophagy can occur when the level of degradation goes beyond the threshold of maintaining the required number of organelles needed for cell survival. Jeong et al. investigated the effects of cannabidiol on oxaliplatin resistance in colorectal cancer cells [3]. They determined that combined cannabidiol with oxaliplatin reduced the phosphorylation of nitric oxide synthase 3 (NOS3), nitric oxide (NO) production, and superoxide dismutase 2 (SOD2) expression resulting in the generation of reactive oxygen species (ROS). Interestingly, the induction of ROS was associated with mitochondrial dysfunction, autophagy, and cell death. In addition, the combination of cannabidiol and oxaliplatin displayed superior anticancer activity as compared with either monotherapy and notably, was able to overcome oxaliplatin resistance. While autophagy induction is frequently associated with cell survival and drug resistance, this study highlights that it can also contribute to cell death under certain circumstances. 

The roles of autophagy in normal and malignant cells can be strikingly different. In normal cells, autophagy functions as a mechanism of tumor suppression by eliminating damaged organelles and proteins to promote cellular homeostasis. However, cancer cells preferentially utilize autophagy to drive metabolic reprogramming that degrades cellular components to generate the necessary energy needed for cell survival during periods of stress such as hypoxia, starvation, and during chemotherapeutic treatment [4]. In addition, basal autophagic activity has been determined to be higher in more advanced and metastatic tumors [5]. Ieni et al. analyzed advanced tubular gastric adenocarcinomas and measured the levels of the autophagy-related proteins microtubule-associated protein 1 light chain 3 (LC3A/B), Beclin-1, and activating molecule in Beclin-1-regulating autophagy protein-1 (AMBRA-1) by immunohistochemistry [6]. Immunostaining demonstrated that LC3A/B, Beclin-1, and AMBRA-1 were selectively expressed in tumor tissue and not in adjacent normal stromal cells. In addition, an autophagy-positive expression signature was associated with poorer overall survival in these patients. Collectively, the authors concluded that autophagy is associated with more aggressive advanced tubular gastric adenocarcinomas. 

Most studies have focused on inhibition of autophagy at the distal point in the process through interference of lysosomal degradation using drugs such as hydroxychloroquine (HCQ) [7]. Indeed, HCQ and the related drug chloroquine (CQ) are the only autophagy inhibitors that have been evaluated in clinical trials to date. Given that those are very old drugs that were not optimized for autophagy inhibition activity during their initial discovery, there is a tremendous interest in developing new agents that target autophagy more robustly particularly at more proximal points in the pathway as this remains an underexplored strategy. Chen et al. investigated the anti-autophagy effects of MPT0L145, a novel inhibitor of phosphatidylinositol 3-kinase catalytic subunit type 3 (PIK3C3) and fibroblast growth factor receptor (FGFR) [8]. PIK3C3 belongs to the PI3K family of kinases and has been previously shown to be an essential factor that promotes autophagy [9,10]. The authors demonstrated that MPT0L145 perturbs autophagic flux and sensitizes cancer cells to the anticancer agents gefitinib and gemcitabine. This study further highlights that targeting PIK3C3 can overcome drug resistance associated with autophagy induced by chemotherapy. 

In addition to these research articles, several excellent reviews summarizing key aspects of the field were published in this Special Issue. It has been well established that autophagy is an important mediator of therapeutic resistance to diverse classes of anticancer agents. Two outstanding articles discuss the mechanisms underlying autophagy-mediated treatment resistance and strategies to enhance chemosensitization through inhibition of autophagy [11,12]. An additional article specifically focuses on autophagy-driven resistance to histone deacetylase (HDAC) inhibitors [13]. Indeed, autophagy has been demonstrated to be a key resistance mechanism to HDAC inhibitor therapy, which has prompted the clinical evaluation of this therapeutic approach [14,15,16]. Taken together, these articles provide a comprehensive review of autophagy as a drug resistance factor and summarize the robust evidence in the literature that demonstrates that targeting autophagy can improve the anticancer activity of many chemotherapeutic agents.

Besides being a facilitator of drug resistance, upregulation of autophagy has been identified as a contributing factor that accelerates disease progression and metastasis in a multitude of tumor types. Saxena et al. review the roles of autophagy in esophageal squamous cell carcinoma and esophageal adenocarcinoma pathogenesis [17]. They also conclude that the development of novel agents that specifically activate or inhibit autophagy is essential to better understand the role of autophagy in malignant biology and to improve the clinical targeting of this pathway. In addition to disrupting the lysosome with agents such as HCQ, CQ, and ROC-325, upstream components of the autophagy machinery may prove to be viable therapeutic targets [4,18,19]. Some of the potential upstream targets in the cascade include the aforementioned PIK3C3 or vacuolar protein sorting 34 (VPS34) as well as UNC-51-like kinase 1 (ULK1) and autophagy-related gene 4 (ATG4). A particularly interesting target is ATG4, which is reviewed in this issue by Fu et al [20]. ATG4 is required for autophagosome formation and studies have suggested that ATG4 may be a potential anticancer target due to its elevated expression in some cancer types [21]. Another interesting review describes the role of actin during autophagy and the development of drug resistance [22]. Actin has previously been demonstrated to be involved in the formation and maturation of autophagic vesicles during the autophagy process [23,24]. They describe how actin manipulation affects autophagy and highlight potential therapeutic targets in this pathway. These reviews illuminate the complexity of autophagy and underscore the need for new agents to innovatively modulate this pathway by targeting previously unexplored regulators of the process. 

The articles in this Special Issue mesh perfectly with each other to highlight the significance of autophagy as a key mechanism that cancer cells utilize to drive malignant progression and drug resistance. The articles comprehensively discuss the rationale for developing novel autophagy-modulating agents and combining them with standard therapeutic regimens to improve clinical outcomes. They also establish the framework for further studies aimed at delineating the differences between inhibiting autophagy at proximal vs. distal (lysosomal) points. Hopefully, the development of specific and more potent compounds will enable optimized precision targeting of autophagy in future clinical studies.
